# The broad role of Nkx3.2 in the development of the zebrafish axial skeleton

**DOI:** 10.1371/journal.pone.0255953

**Published:** 2021-08-19

**Authors:** Laura Waldmann, Jake Leyhr, Hanqing Zhang, Caroline Öhman-Mägi, Amin Allalou, Tatjana Haitina

**Affiliations:** 1 Department of Organismal Biology, Uppsala University, Uppsala, Sweden; 2 Division of Visual Information and Interaction, Department of Information Technology, Uppsala University, Uppsala, Sweden; 3 Science for Life Laboratory BioImage Informatics Facility, Uppsala, Sweden; 4 Department of Materials Science and Engineering, Uppsala University, Uppsala, Sweden; Laboratoire de Biologie du Développement de Villefranche-sur-Mer, FRANCE

## Abstract

The transcription factor Nkx3.2 (Bapx1) is an important chondrocyte maturation inhibitor. Previous *Nkx3*.*2* knockdown and overexpression studies in non-mammalian gnathostomes have focused on its role in primary jaw joint development, while the function of this gene in broader skeletal development is not fully described. We generated a mutant allele of *nkx3*.*2* in zebrafish with CRISPR/Cas9 and applied a range of techniques to characterize skeletal phenotypes at developmental stages from larva to adult, revealing loss of the jaw joint, fusions in bones of the occiput, morphological changes in the Weberian apparatus, and the loss or deformation of bony elements derived from basiventral cartilages of the vertebrae. Axial phenotypes are reminiscent of *Nkx3*.*2* knockout in mammals, suggesting that the function of this gene in axial skeletal development is ancestral to osteichthyans. Our results highlight the broad role of *nkx3*.*2* in zebrafish skeletal development and its context-specific functions in different skeletal elements.

## Introduction

NK3 homeobox 2 (Nkx3.2, Bapx1) is an evolutionarily conserved gene encoding a homeodomain-containing transcription factor that is involved in cartilage growth and differentiation in gnathostomes. It was first described in *Drosophila* (*bagpipe*, *bap*), where it plays a major role in the visceral mesoderm during the formation of the midgut musculature [1]. During vertebrate evolution *Nkx3*.*2* expression was incorporated into the intermediate domain of the first pharyngeal arch. This event has been proposed to be crucial for jaw joint formation during the transition from jawless to jawed vertebrates [2]. Jawed vertebrates like the zebrafish, frog, and chicken display a focal expression of *Nkx3*.*2* between Meckel’s and palatoquadrate cartilages of the first pharyngeal arch skeleton [3–5]. In contrast, *Nkx3*.*2* expression in the jawless lamprey is not limited to the first arch, instead showing a broader expression in the anterior pharyngeal ectoderm and endoderm [2,6]. Endothelin-1 signaling through its receptor directs the dorsoventral patterning of the migrating neural crest cells that form the skeleton of the first pharyngeal arch [3,7]. Previous studies showed that Nkx3.2 is an essential factor for primary jaw joint development, as the loss of expression in zebrafish, frog, and chick leads to a failure in jaw joint formation accompanied by the fusion of the joint articulating cartilage elements: Meckel´s cartilage and the palatoquadrate [3,5,8]. Two recently described *nkx3*.*2* null alleles caused by frameshift mutations demonstrated ankylosis in zebrafish that could survive to adulthood [9]. Overexpression of *nkx3*.*2* (*bapx1*) in amphibians induces the formation of ectopic cartilage elements by introducing additional subdivisions into existing cartilage, clearly showing the joint-promoting effect of this transcription factor [10].

In the course of early Mammalian evolution, the primary jaw joint between the articular and quadrate bones was incorporated into the middle ear to form the incudomalleolar joint, and a new jaw joint was formed between the dentary and squamosal [11,12]. As a consequence, *Nkx3*.*2* is expressed within the middle ear-associated bones of the tympanic ring and gonium as well as in the incudomalleolar joint in mammals [13]. Further expression analysis in mouse embryos showed *Nkx3*.*2* expression in the developing vertebrae and the cartilaginous condensations of the developing limbs [14]. Mouse embryos deficient in *Nkx3*.*2* display hypoplasia in the tympanic ring, the absence of the gonium, a size reduction of cranial occipital bones such as the basioccipital and basisphenoid, and finally the loss of the supraoccipital bone and vertebral ossification centres [13,15,16]. Various studies describe Nkx3.2 as a chondrocyte maturation inhibitor during chondrogenesis [17–20]. In chicken and mouse long bone development, Nkx3.2 can repress the chondrocyte maturation factor Runx2 during endochondral ossification and thus maintains the chondrocytes in an immature state [19]. In humans homozygotic mutations in NKX3.2 leads to a spondylo-megaepiphyseal-metaphyseal dysplasia (SMMD), a rare skeletal disease [21,22]. The patients suffer from, among other symptoms, a short stature, stiff neck and trunk, and defects in vertebral ossification [21–25], similar to what was observed in mouse knockout mutants. These data clearly indicate a role of Nkx3.2 in the mammalian axial skeleton beyond just the middle ear that is homologous to the non-mammalian primary jaw joint. However, the function of this gene in the axial skeleton of non-mammals has been investigated very briefly [26]. In zebrafish embryos and juveniles *nkx3*.*2* expression can be detected in the jaw joint, around the anterior notochord and vertebrae, and in the proximal radials of the median fins [27,28].

In this study, we present a comprehensive investigation of the function of *nkx3*.*2* in zebrafish skeletal development by generating a CRISPR/Cas9 induced mutant line and characterizing larval, juvenile, and adult phenotypes. Our results confirm and elaborate on the primary jaw joint loss reported in previous studies and we describe novel axial phenotypes in the occiput, Weberian apparatus, and rib-bearing vertebrae, pushing back the likely origin of axial functions of Nkx3.2 to the osteichthyan stem group.

## Materials and methods

### Ethical statement

All animal experimental procedures were approved by the local ethics committee for animal research, Uppsala djurförsöksetiska nämnd (permit numbers C161/4 and 5.8.18-18096/2019). All procedures for the experiments were performed in accordance with the Swedish Board of Agriculture’s Regulations and General Advice about Laboratory Animals.

### CRISPR/Cas9 target design

Two sgRNAs targeting the single zebrafish *nkx3*.*2* gene with no predicted off-target effects were designed using the online software CHOPCHOP [29], both targeting the first exon: 5´-GATCAGGAATCCGCGGCCAA-3´ and 5´-GTCGTTGTCCTCGCTCAGCC-3´. The second base of each target was modified to "G" in order to allow T7 transcription without modifications. The sgRNAs were prepared as previously described [30], creating a fragment consisting of the T7 promotor, the targeted gene-specific sequence, and the guide core sequence. The sgRNAs were synthesised by in vitro transcription using the HiScribe T7 High Yield RNA Synthesis Kit (New England Biolabs, Ipswich, MA). Cas9 mRNA was prepared by in vitro transcription with the mMESSAGE mMACHINE T3 Transcription Kit (Life Technologies, Carlsbad, CA) using 500 ng of linearised plasmid that was retrieved from 5 μg of p-T3TS-nCas9n plasmid (plasmid #46757; Addgene, Cambridge, MA) digested with XbaI (New England Biolabs, Ipswich, MA). The gRNAs were purified with mirVana miRNA isolation kit (Life technologies, Carlsbad, CA) and Cas9 mRNA was purified with RNeasy Mini Kit (Qiagen, Maryland; MD), and their integrity was assessed using a denaturation gel.

### Generation of zebrafish mutant line

Fertilised zebrafish (*Danio rerio*) eggs were obtained by natural spawning of *Tg(sox10*:*egfp)* line [31]. Embryos were injected at the one-cell stage with 150 pg of Cas9 mRNA and 50 pg of each sgRNA in RNase-free water as previously described [30], and maintained at 28.5°C in E3 medium [32]. The efficiency of the targets was estimated by the CRISPR-Somatic Tissue Activity Test (STAT) methodology in eight embryos at two days post-injection, as previously described [33]. The injected founder zebrafish (F0) were raised and incrossed. For genotyping the F1 zebrafish, DNA was extracted from a 1–3 mm amputation of the adult zebrafish caudal fin by lysing the tissue in 30 μl of 50 mM NaOH for 20 min at 95°C, adding 60 μl of 0.1 mM Tris and diluting the obtained material (1:10). For the initial genotyping step, FLA analysis was used. 2 μl of DNA (50–200 ng) was added to Platinum Taq DNA Polymerase. The PCR mix was incubated at 94°C for 12 min followed by 35 cycles of 94°C for 30 sec, 57°C for 30 sec, 72°C for 30 sec, and final extension at 72°C for 10 min. Size determination was carried out on a 3130XL ABI Genetic Analyzer (Applied Biosystems, Waltham, MA) and the data were analysed using the Peak Scanner Software (Thermo Fisher Scientific, Waltham, MA). For the fish that screened positive for the variant, the FLA results were confirmed by Sanger sequencing.

### Founder screening and identification of heterozygous adult fish

sgRNA for both targets were co-injected and we have genotyped the fish for potential mutations at both target sites. One strain with an allele containing a frameshift deletion resulting in a premature stop codon (*nkx3*.*2*^uu2803^) was selected for further experiments. The identified F1 founders were crossed with wild-type zebrafish (AB strain), and their adult offspring (F2) were genotyped. Heterozygous F2 fish of mutant line *nkx3*.*2*^uu2803^ were incrossed and the offspring was observed with bright-field and fluorescence microscopy. Embryos showing phenotypes were euthanised with an overdose of MS-222 (300mg/L) and genotyped by FLA and/or Sanger sequencing (forward primer: 5’-TGTAAAAC-GACGGCCAGTGAGGAGTCTCGCCATCTGAA-3’; reverse primer: 5’-GTGTCTTACAGATGAA-GCTTTGAGTGGT-3’).

### *In vivo* microscopy

Fluorescent images were obtained with an inverted Leica TCS SP5 confocal microscope using LAS-AF software (Leica Microsystems). Embryos were sedated with 0.16% MS-222 and embedded in 0.8% low melting agarose onto the glass bottom of the 35mm dishes. To prevent drying, embedded embryos were covered with system water containing 0.16% MS-222. Screening for GFP in zebrafish larvae was performed using a Leica M205FCA fluorescence microscope with the appropriate filter.

### Histological analysis

Zebrafish juveniles and adults at 14, 30 and 90 dpf were euthanised with an overdose of MS-222 (300mg/L), fixed in 4% PFA and washed in PBST buffer. 30 and 90 dpf fish were decalcified in 0.5 M EDTA for one week with EDTA exchange every third day. Fish were transferred into 99.5% ethanol, followed by Xylene and embedded into paraffin. Sagittal head sections of 6μm were prepared with Leica RM2155 Rotary Microtome. Tissue sections were deparaffinised with xylene and re-hydrated through 99.5% to 70% ethanol series and transferred to water. Sections were then stained with Nuclear Fast Red (Vector Laboratories, Burlingame, CA) for 30 sec followed by a brief water rinse and dehydration in 95% and 99.9% ethanol. Prior to mounting with VectaMount (Vector Laboratories, Burlingame, CA), slides were washed in Clear-Rite 3 (Richard-Allan Scientific, Kalamazoo, MI) three times for three minutes each. Sections were imaged with 40x objective on Hamamatsu NanoZoomer S60 Digital Slide Scanner.

### Skeletal staining

Staining of cartilage and bone was done based on the previously published protocol by Walker and Kimmel [34]. Zebrafish wild-type and mutant fish at 5, 9, 14 and 30 dpf, were euthanised with an overdose of MS-222 (300mg/L), fixed in 4% PFA and transferred to 50% ethanol. For cartilage staining, specimens were immersed in alcian blue solution (0.02% Alcian Blue 8 GX, 50mM MgCL2, 70% ethanol), and for bone staining, specimens were immersed in alizarin red solution (0.5% Alizarin Red S). For double staining of cartilage and bone, specimens were immersed in double staining solution (99% alcian blue solution, 1% alizarin red solution). After staining overnight, specimens were washed twice with 50% ethanol and then immersed in water for 2 hours before being bleached in a solution of 1.5% H202 and 1% KOH until pigmentation was removed. 30 dpf specimens were then immersed in trypsin solution (1% trypsin, 35% sodium tetraborate) for 30 minutes followed by incubation in a solution of 10% glycerol and 0.5% KOH for 1 hour. All specimens were imaged with a Leica M205FCA microscope in a solution of 50% glycerol and 0.25% KOH, followed by storage in 50% glycerol and 0.1% KOH.

### Optical projection tomography

A custom-built Optical Projection Tomography (OPT) system was used for imaging of the zebrafish embryos at 5 dpf that were euthanised with an overdose of MS-222 (300mg/L), fixed and stained with alcian blue [35,36]. The OPT system, reconstruction algorithms, and alignment workflow were based on the previously described method [37]. All embryos were kept in 99% glycerol before they were loaded into the system for imaging. The rotational images were acquired using a 3X telecentric objective with a pixel resolution of 1.15 μm/pixel. The tomographic 3D reconstruction was done using a filtered back projection (FBP) algorithm in MATLAB (Release R2015b; MathWorks, Natick, MA) together with the ASTRA Toolbox [38]. For the data alignment, the registration toolbox elastix [39,40] was used. To reduce the computational time all 3D volumes in the registration were down-sampled to half the resolution.

The registration workflow was similar to the methods described in ref. [37] where the wildtype fish were initially aligned and used to create an average reference fish using an Iterative Shape Averaging (ISA) algorithm [41]. All wild-type (*n* = 10) and *nkx3*.*2*^uu2803/uu2803^ (*n* = 11) zebrafish were then aligned to the reference. After the alignment, a voxel-wise method was used to detect voxels that are significantly different between the groups. The Mann-Whitney U test was used to compare corresponding voxels in wild-type and mutant. The p-value threshold is set using a false discovery rate (FDR) [42] and a permutation test [43]. The FDR was set so that those random groupings showed only a small number of significant voxel differences (p<2.5x10^-4^; FDR = 0.045). All registration and analysis were done on the green channel of the RGB images.

### Micro-computed tomography and segmentation

Five wild-type and five *nkx3*.*2*^uu2803/uu2803^ zebrafish at 90 dpf were euthanised with an overdose of MS-222 (300mg/L), fixed, and analysed with micro-computed tomography (μCT, SkyScan 1172, Bruker microCT, Belgium) at a voltage of 60 kV, a current of 167 μA, and an isotropic voxel size of 5.43 μm. Cross-sections were reconstructed using software package NRecon (NRecon 1.6.10, Bruker microCT, Belgium). The specimens were placed in 2mL Eppendorf tubes filled with 1% agarose and the 10ul pipette tip was used to keep the mouths of some wild-type fish in the open position. BMP image stacks obtained with μCT were imported into, segmented, and imaged using VGStudio MAX version 3.2.5 (Volume Graphics, Germany).

## Results

### Zebrafish *nkx3*.*2*^uu2803/uu2803^ line generated with CRISPR/Cas9 survives to adulthood and has open mouth phenotype

**Fig 1:**
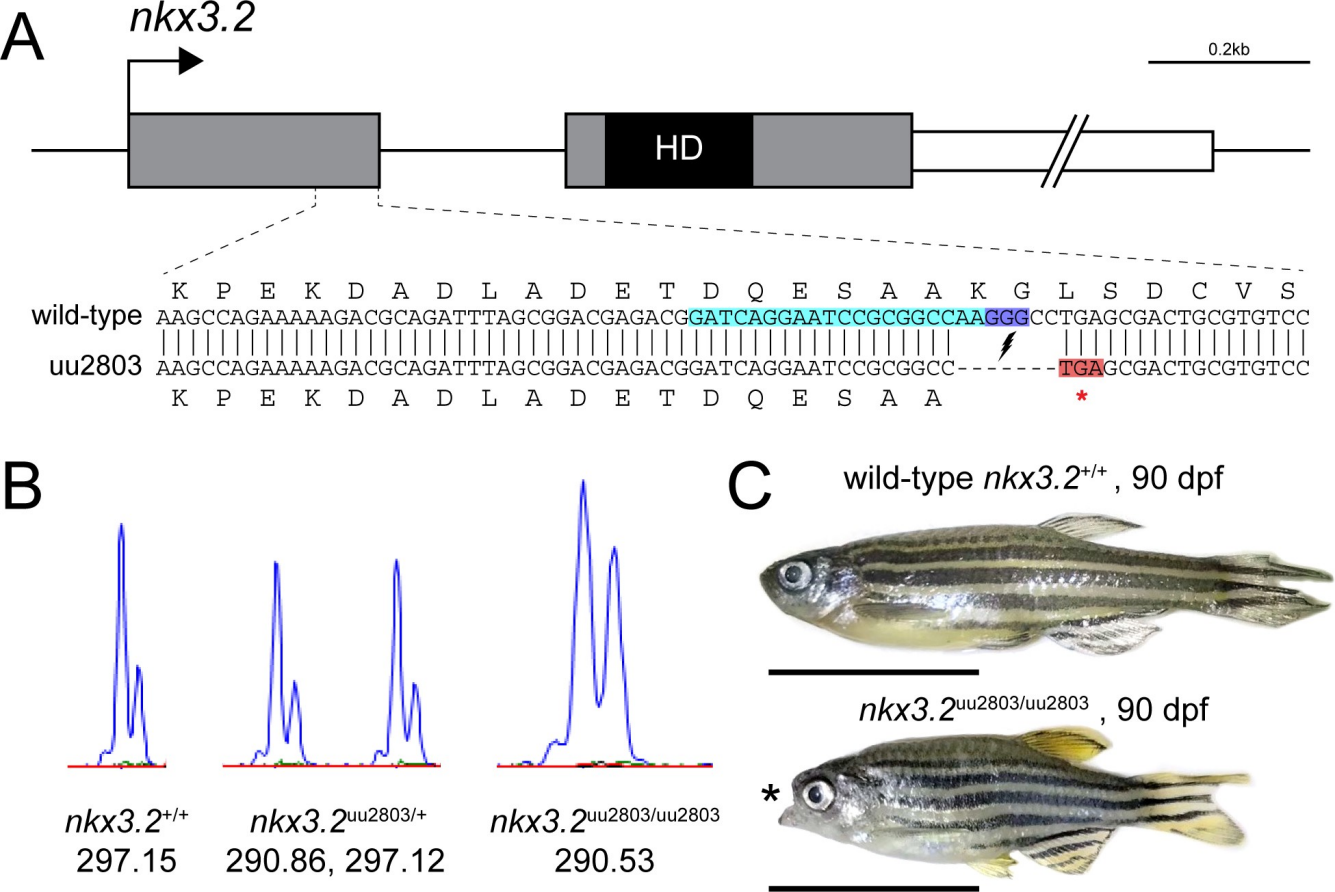
Zebrafish *nkx3*.*2* null allele generated with CRISPR/Cas9. (**A**) To-scale schematic of the two-exon zebrafish *nkx3*.*2* gene locus on chromosome 14. Grey boxes represent the coding sequence, the black box labelled “HD” marks the location of the homeodomain-coding sequence in the second exon, and the white box marks the 1,226bp 3’ UTR, truncated for illustrative purposes. gRNA target site is marked by turquoise background. The sequence of the zoomed in section of exon 1 shows the alignment between the wild-type and mutant (uu2803) alleles. The mutant allele has a 7 base-pair deletion that results in a frameshift and a premature stop codon (highlighted in red) immediately after the deletion site. (**B**) Wild-type, heterozygous *nkx3*.*2*^uu2803/+^ and homozygous *nkx3*.*2*^uu2803/uu2803^ fish were genotyped by fragment length analysis. The wild-type displayed one peak (297,15), *nkx3*.*2*^uu2803/+^ fish one wild-type (297,12) and one mutant (290,86) peak and *nkx3*.*2*^uu2803/uu2803^ fish one mutant peak (290,53). (**C**) *nkx3*.*2*^uu2803/uu2803^ fish at 90 dpf display a prominent fixed open mouth phenotype compared to wild-type at 90 dpf. Scale bars: 1 cm.

Clustered Regulatory Interspaced Short Palindromic (CRISPR)/CRISPR-associated protein 9 (Cas9) was used to generate *nkx3*.*2* mutant line to analyse both embryonic and adult mutant phenotypes. Both of the designed sgRNAs showed similar activity and during the genotyping step we selected founder fish with a frameshift mutation caused by one of the sgRNAs (Fig 1A). We generated a mutant allele with a 7 bp deletion in *nkx3*.*2* exon 1 (c.286_292 del, p.Lys95*), causing a frameshift, which resulted in a premature stop codon that shortens the peptide sequence to 95 amino acids, compared to 245 amino acids in the wild-type (Fig 1A). The last eight amino acids encoded by the first exon and all amino acids encoded by the second exon including the entire DNA-binding homeodomain of Nkx3.2 are predicted to be absent in this shortened protein and these changes generate a severe loss-of-function allele.

Heterozygous embryos (*nkx3*.*2*^uu2803/+^) displayed no gross morphological differences compared to wild-type (*nkx3*.*2*^+/+^) embryos as long as observed. Homozygous mutant embryos displaying morphological differences were generated by crossing two heterozygous *nkx3*.*2*^uu2803/+^ adult zebrafish. Homozygous mutants (*nkx3*.*2*^uu2803/uu2803^) were able to survive to adulthood and displayed a prominent fixed open mouth phenotype (n = 6/6, Fig 1C). Adult homozygous mutants (further in the text referred to as mutants) at 90 dpf displayed no significant differences in standard length (SL) relative to adult wild-types, although they showed a rather wide distribution of SL (S1 Fig).

The differences in SL between wild-type and mutant groups were apparent at 30 dpf and 60 dpf. Possible explanations are that bigger SL in mutants at 30 dpf was due to overeating caused by constant swimming through food with an open mouth, whereas smaller SL at 60 dpf was due to some competition for food before separation of the mutant group at 60 dpf (S1 Fig). However, other explanations such as differences in tank densities or cryptic genetic variation are also plausible, and we cannot rule them out.

### Confocal live imaging displays fusion of Meckel’s cartilage with palatoquadrate in *nkx3*.*2*^uu2803/uu2803^ zebrafish embryo and larvae

**Fig 2:**
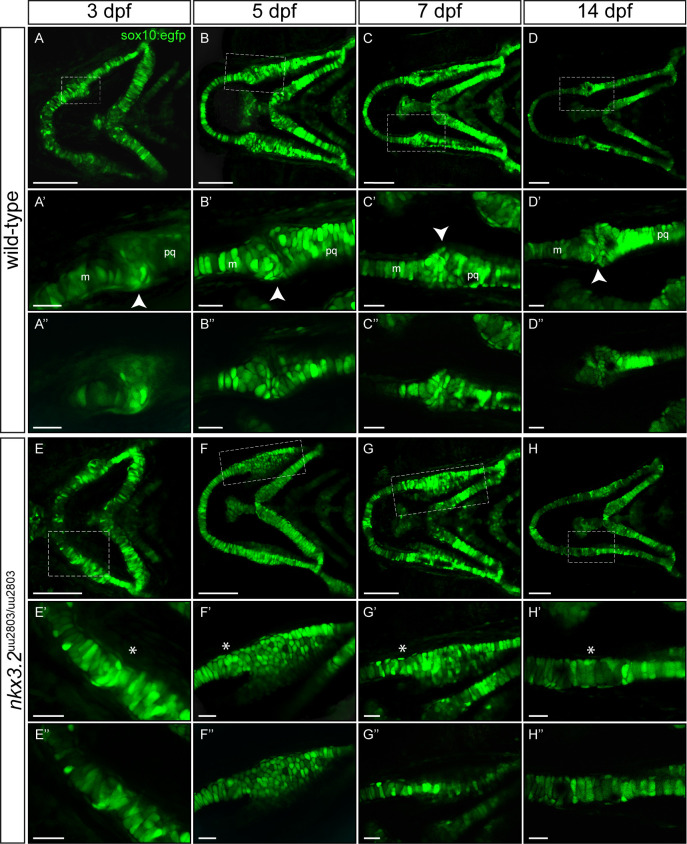
Larval development of wild-type and *nkx3*.*2*^uu2803/uu2803^ jaw joints. (**A-H’**) Maximum projections of confocal live imaging Z-stacks acquired from ventral side of wild-type zebrafish head at 3 dpf (**A, A’**), 5 dpf (**B, B’**), 7dpf (**C, C’**), 14 dpf (**D, D’**) and *nkx3*.*2*^uu2803/uu2803^ zebrafish head at 3 dpf (**E, E’**), 5 dpf (**F, F’**), 7 dpf (**G, G’**), 14 dpf (**H, H’**) in Tg(*sox10*:*egfp*) background. Corresponding single Z-planes are shown in **A”–H”**. (**A-D**´´) The jaw joint in wild-type zebrafish (dashed box) is magnified in **A’-D”.** The retroarticular process is visible from 3 dpf onwards, marked by white arrowhead. Chondrocytes of the anterior Meckel’s cartilage and posterior palatoquadrate align in stacks. Posterior Meckel’s cartilage and anterior palatoquadrate articulate the jaw joint. (**E-H”**) Fusion of jaw joint articulating elements in *nkx3*.*2* mutants. The fusion site is magnified in (**E’-H”)** and indicated by asterisks. (**E’-F’** and **E”-F”**) At 3 dpf and 5 dpf *nkx3*.*2* mutants display unorganised and rounded cells in the anterior palatoquadrate, posterior to the jaw joint fusion site (**G’, G”**) At 7 dpf *nkx3*.*2* mutants display elongated chondrocytes that start to align in stacks. (**H’, H”**) At 14 dpf *nkx3*.*2* mutants display elongated chondrocytes that are aligned throughout the fused Meckel’s-palatoquadrate element. m—Meckel’s cartilage, pq—palatoquadrate. Scale bars: 100 μm (**A-H**), 25 μm in (**A’-H’** and **A”-H”**).

In order to follow the first mandibular arch development, confocal live imaging was performed using a transgenic *sox10*:*egfp* line labelling neural crest-derived cells of the pharyngeal arches. Clear phenotypic differences in the jaw joint-forming region were detectable from 3 dpf onwards (Fig 2, S1 Movie). The jaw joint was lost in *nkx3*.*2*^uu2803/uu2803^ fish as Meckel’s cartilage and the palatoquadrate were fused from 3 dpf onwards (n = 4/4, Fig 2E–2H”) and the retroarticular process (RAP) was missing at the posteroventral tip of Meckel’s cartilage (n = 4/4, Fig 2E–2H”, S1 Movie). *nkx3*.*2* mutants furthermore showed an unorganised cell-mass of small rounded chondrocytes posterior to the jaw joint fusion site, in the anterior part of the palatoquadrate at 3–5 dpf (n = 4/4, Fig 2E–2F”). By 7dpf chondrocyte of the anterior palatoquadrate were elongated and displayed typical stacking (n = 4/4, Fig 2G–2G”, S1 Movie). At 14 dpf, all chondrocytes were elongated and displayed alignment throughout the entire Meckel’s–palatoquadrate fused element (n = 3/3, Fig 2H–2H”, S1 Movie).

### Histological analysis of *nkx3*.*2*^uu2803/uu2803^ zebrafish shows chondrocyte alignment and hypertrophy at the jaw joint fusion site

**Fig 3:**
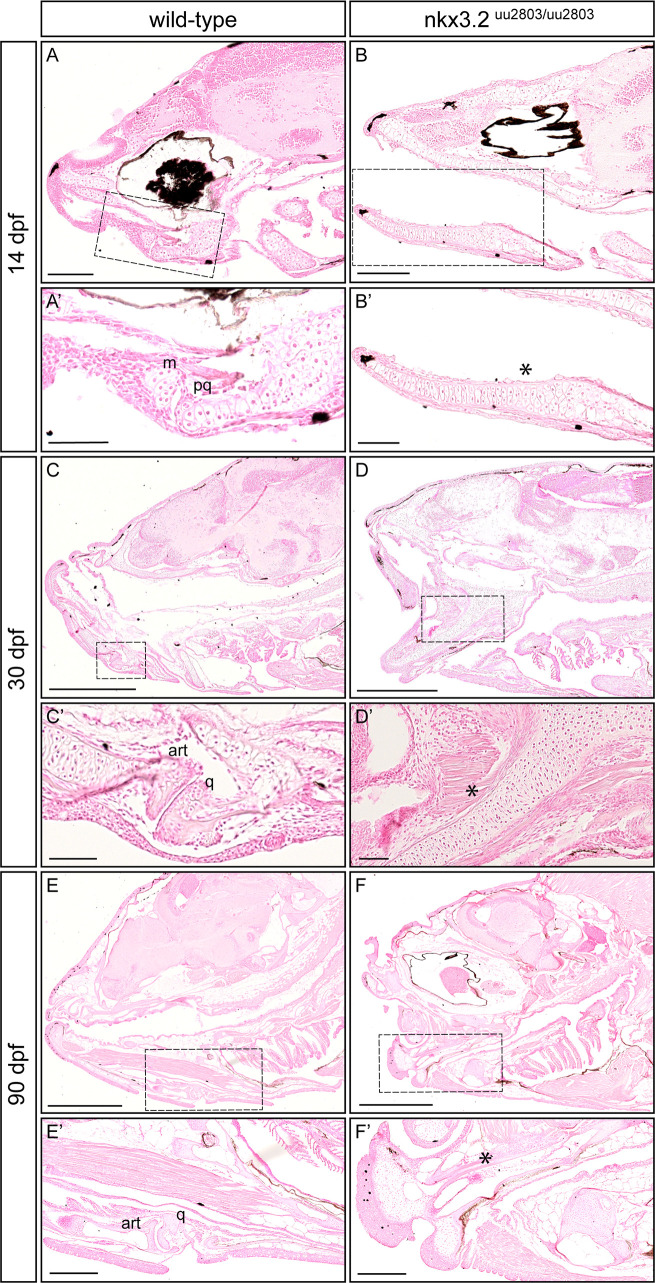
Histological analysis reveals loss of jaw joint without affected chondrocyte hypertrophy in the first pharyngeal arch of *nkx3*.*2*^uu2803/uu2803^ zebrafish. Sagittal sections of wild-type 14 dpf (**A, A’**), 30 dpf (**C, C’**) and 90 dpf (**E, E’**) and *nkx3*.*2* mutant 14 dpf (**B, B’**) 30 dpf (**D, D’**) and 90 dpf (**F, F’**) zebrafish stained with Nuclear Red. (**A, A’, C, C’, E, E’**) Stained histology sections displaying a normal jaw joint development between Meckel’s cartilage (m) and palatoquadrate (pq), respectively articular (art) and quadrate (q) in wild-type fish. (**B, B’, D, D’, F, F’**) *nkx3*.*2* mutants do not display a jaw joint. Chondrocyte maturation and ossification seem not to be affected in *nkx3*.*2*^uu2803/uu2803^ besides the absence of joint-typical cells lining the articulating elements. Dashed box in (**A-F)** marks the magnified region in (**A’-F’)**. Asterisk marks the fused element. m–Meckel’s cartilage, pq–palatoquadrate, art–articular, q–quadrate. Scale bars: 200μm (**A-F**), 100μm (**A’-F’**).

Histological sections were prepared to further analyse the chondrocyte arrangement within the first mandibular arch in *nkx3*.*2* mutant larval, juvenile, and adult zebrafish. A joint gap between Meckel´s cartilage and the palatoquadrate is visible in wild-type zebrafish at 14 dpf (Fig 3A and 3A’). In wild-types at 14 dpf, articular chondrocytes line the joint cavity and hypertrophic chondrocytes form the articulating elements (Fig 3A and 3A’). In *nkx3*.*2* mutant larvae at 14 dpf, Meckel´s cartilage and the palatoquadrate were not separated but fused–the jaw joint was absent (Fig 3B and 3B’). Chondrocytes within the fused element were hypertrophic and aligned. At the presumptive fusion site, the element appeared to be increased in width caused by piled-up rows of aligned chondrocytes (Fig 3B and 3B’). The exact fusion point was difficult to determine in both 30 dpf and 90 dpf mutants as we did not see any clear border between articular and quadrate (n = 7/7; Fig 3D, 3D’, 3F and 3F’), but apart from the fusion and its phenotypic consequences, ossification seemed not to be affected. These results are different from Miyashita *et al*. [9] who showed a clear boundary between articular and quadrate bones. However, we cannot exclude that a very faint border could exist in our mutants, but was not contrasted with Nuclear Red staining. Articular chondrocytes lining the articulating tips of the articular and quadrate were consequently absent in *nkx3*.*2* mutant juvenile fish at 30 dpf (Fig 3D and 3D’). Chondrocytes within the fused element displayed hypertrophic morphology similar to wild-type (Fig 3C–3D’). By 90 dpf, ossification of the fused articular and quadrate was completed, visible by the presence of adipose tissue inside the bones in both wild-type and mutant adult fish (Fig 3E–3F’).

### Optical projection tomography reveals morphological changes in the head of *nkx3*.*2*^uu2803/uu2803^ larvae

**Fig 4:**
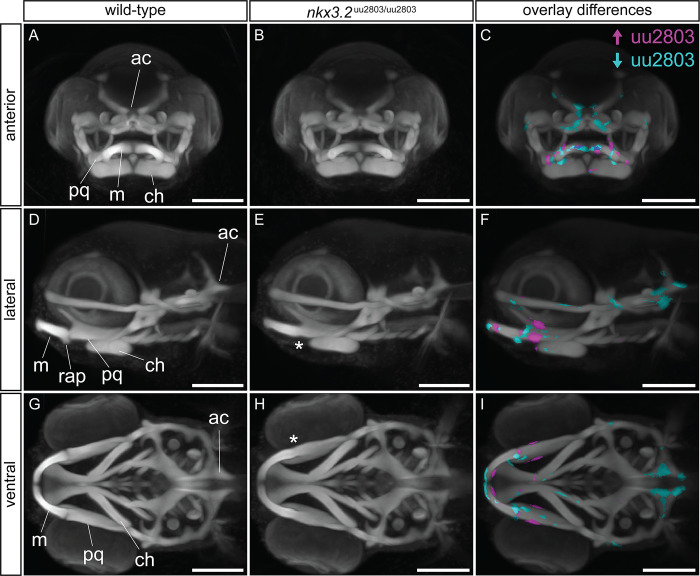
Optical projection tomography of cartilage-stained wild-type and *nkx3*.*2*^uu2803/uu2803^ larvae reveals subtle phenotypic changes. (**A, D, G**) Maximum projection of 5 dpf wild-type group (n = 10). (**B, E, H**) Maximum projection of 5 dpf *nkx3*.*2* mutant (n = 11) group. (**C, F, I**) Maximum projection of both groups with coloured voxels representing voxels with statistically significant (p<2.5x10^-4^) differences in intensity. Magenta shows voxels with higher intensity and cyan shows voxels with lower intensity in *nkx3*.*2* mutant group. Asterisk marks the fusion site. ch–ceratohyal, m–Meckel’s cartilage, ac–auditory capsule, pq–palatoquadrate, rap–retroarticular process. Scale bars: 150μm.

In order to characterise the larval *nkx3*.*2* mutant phenotype in greater detail, we used optical projection tomography (OPT) on 5 dpf cartilage-stained wild-type (n = 10) and mutant (n = 11) larvae to reconstruct 3D models of cartilage morphology. Multiple wild-type (n = 10) and mutant (n = 11) 3D models were overlaid and combined to produce an average wild-type morphology (Fig 4A, 4D and 4G) and an average *nkx3*.*2* mutant morphology (Fig 4B, 4E and 4H), respectively. Overlaying these grouped 3D reconstructions allowed the calculation of voxels that were statistically more intense or less intense in the mutant group (Fig 4C, 4F and 4I), corresponding to locations with more or less cartilage present, respectively. This analysis was robust to false positives (S2 Fig).

The *nkx3*.*2* mutant group showed significantly higher intensity of the cartilage labelling at the jaw joint region (magenta in Fig 4F and 4I) consistent with the presence of fused cartilage in mutants compared to the jaw joint gap in wild-type larvae. Cyan voxels ventral to the jaw joint in Fig 4F indicate the absence of the RAP. The palatoquadrate displayed significantly higher intensity in *nkx3*.*2* mutant group consistent with the increased thickness of this element (magenta in Fig 4C, 4F and 4I). The anterior part of Meckel’s cartilage displayed an increased posterior intensity and decreased anterior intensity in the *nkx3*.*2* mutant group indicating a change in the shape and increased thickness of Meckel’s cartilage (Fig 4I). Interestingly, this analysis also revealed significantly reduced cartilage staining signal in the posterior part of the head, around the auditory capsule (Fig 4F and 4I). The mutant group clearly showed the shorter parts of the posterior auditory capsule changing the shape of the notochord insertion area, compared to the wild-type group (Fig 4F and 4I).

### Cartilage and bone staining analysis of wild-type and *nkx3*.*2*^uu2803/uu2803^ larval, juvenile and adult zebrafish

**Fig 5:**
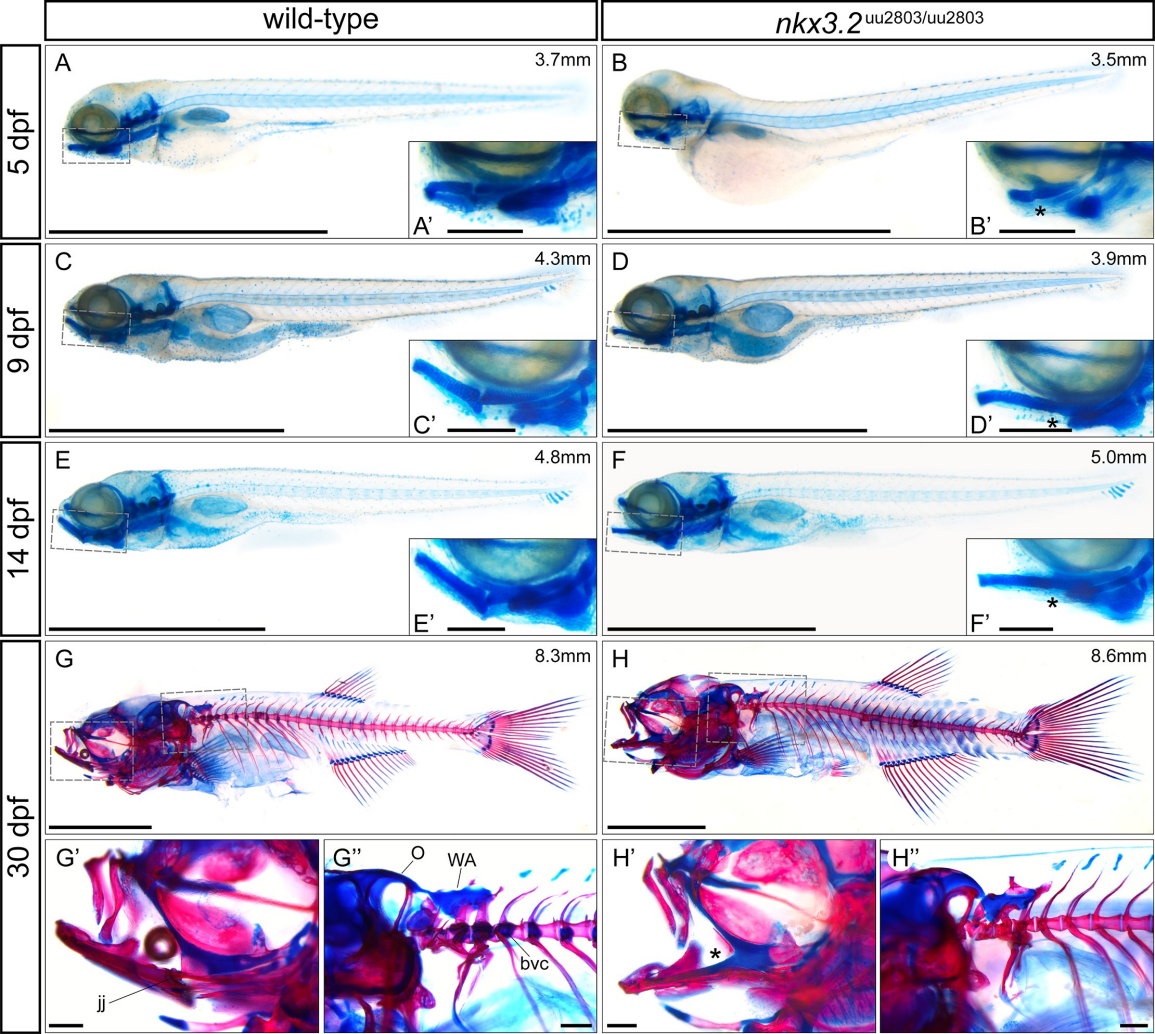
Skeletal staining of wild-type and *nkx3*.*2*^uu2803/uu2803^ zebrafish. (**A-F**) lateral views of cartilage-stained wild-types and *nkx3*.*2* mutants at 5, 9 and 14 dpf. (**G-H**) lateral views of cartilage- and bone-stained wild-types and *nkx3*.*2* mutants at 30 dpf. Boxes in (**A-H**) indicate the zoomed-in regions in the insets or zoomed-in panels (**G’**, **G”**, **H’**, **H”**). The measurements in mm refer to standard length (SL). Asterisks indicate the fusion between Meckel’s cartilage and palatoquadrate in the jaw joint. jj–jaw joint, O–occipital region, bvc–basiventral cartilage, WA–Weberian apparatus. Scale bars: 2mm (**A**-**H**), 200μm (**A’-H’** and **G”-H”**).

Skeletal staining of larval, juvenile and adult *nkx3*.*2* mutant zebrafish was performed to analyse both cartilage and bone abnormalities in comparison to wild type at a greater range of developmental stages (Fig 5). The loss of the jaw joint caused by the fusion of Meckel´s cartilage and palatoquadrate was clearly visible from 5 dpf onwards and was most recognizable by the absence of the RAP (Fig 5B, 5D and 5F). The resulting open mouth phenotype could be observed from 9 dpf onwards (Fig 5D, 5F and 5H). No other cartilage phenotypes within the pharyngeal arches could be detected at any developmental stage, consistent with the OPT results at 5 dpf in Fig 4. Ossification of the dentary, articular, and quadrate appeared to take place normally in *nkx3*.*2* mutants at 30 dpf (n = 2/2, Fig 5H and 5H’) when compared to wild-type fish (Fig 5G and 5G’).

### μCT reveals craniofacial phenotypes in adult *nkx3*.*2*^uu2803/uu2803^ zebrafish

**Fig 6:**
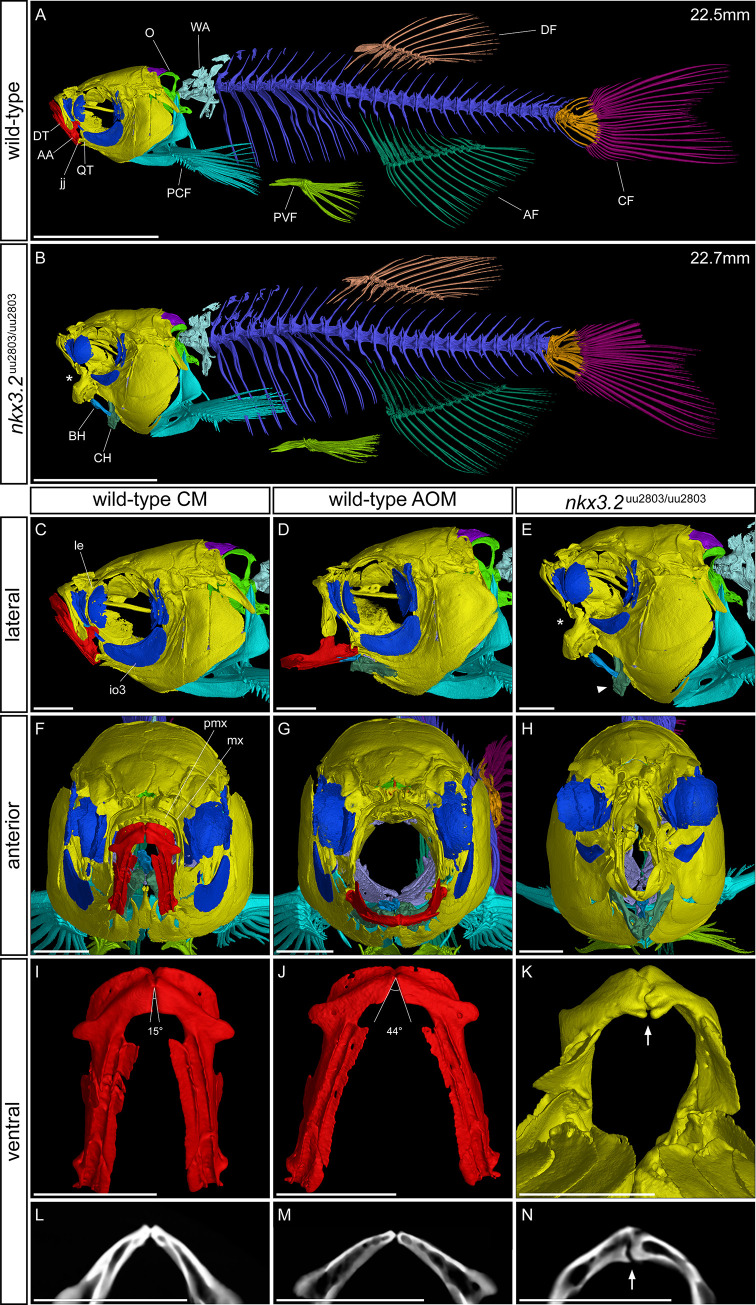
μCT reveals craniofacial phenotypes in adult wild-type and *nkx3*.*2*^uu2803/uu2803^ zebrafish. (**A, B**) lateral view of wild-type and *nkx3*.*2* mutant zebrafish at 90 dpf. (**C, D, E**) lateral view of the head of wild-type with closed mouth (CM), wild-type with artificially open mouth (AOM), and *nkx3*.*2* mutant. (**F, G, H**) Anterior view of wild-type with CM, wild-type with AOM, and *nkx3*.*2* mutant. (**I, J, K**) ventral view of isolated wild-type dentary (CM), wild-type dentary (AOM), and *nkx3*.*2* mutant dentary. (**L, M, N**) Virtual thin sections (60μm thick, averaged) through the symphysis of the dentaries displayed in **I, J, K**, respectively. The measurements in mm refer to standard length (SL). Asterisks in (**B**, **E**) indicate the fixed open mouth phenotype caused by fused jaw joint. The arrowhead in (**E**) indicates the downturned ceratohyal phenotype relative to (**D**). Angles in (**I**, **J**) show the flexibility of the symphysis in wild-types, the arrow in **K** and **N** indicates the thickened and deformed symphysis phenotype in *nkx3*.*2* mutants. Colour scheme: Red–lower jaw; dark blue–infraorbitals; dark green–ceratohyal and anal fin; blue–basihyal; lilac–branchial arches; dark purple–supraoccipital; lime green–exoccipital and basioccipital; yellow–all remaining craniofacial bones; cyan–cleithrum and pectoral fins, arctic blue–Weberian apparatus; blue violet–vertebrae; green–pelvic fins; bronze–dorsal fin; orange–caudal fin vertebrae and hypurals; magenta–caudal fin rays. AA–anguloarticular, AF–anal fin, BH–basihyal, CF–caudal fin, CH–ceratohyal, DF–dorsal fin, DT–dentary, io3 –infraorbital 3, jj–jaw joint, le–lateral ethmoid, mx–maxilla, O–occipital region, PCF–pectoral fin, pmx–premaxilla, PVF–pelvic fin, QT–quadrate, WA–Weberian apparatus. Scale bars: 5mm (**A**, **B**), 1mm (**C**-**N**).

In order to examine the skeleton of adult zebrafish we performed μCT followed by 3D reconstruction of wild-type and *nkx3*.*2* mutant zebrafish at 90 dpf (Fig 6). The resting position of the mouth is closed (CM) in wild-type individuals (Fig 6A, 6C and 6F), so to be better able to compare the phenotype of the wild-type to the fixed open mouth phenotype of the mutant, μCT scans were also performed on 90 dpf zebrafish with their mouths held open (AOM in Fig 6D and 6G) with a pipette tip which produces an unnatural but reasonable approximation of the appearance of the zebrafish head skeleton with an open mouth.

The fusion of the Meckel’s and palatoquadrate cartilages early in the development of *nkx3*.*2* mutants effectively contributed to a fusion of the articular and quadrate that ossify from these cartilage precursors. This made it extremely challenging to demarcate the articular and quadrate in the *nkx3*.*2* mutants at 90 dpf, which is why the lower jaw was not segmented in red in Fig 6B, 6E, 6H and 6K. As in younger *nkx3*.*2* mutants (Fig 5D, 5F and 5H), the mouth of 90 dpf mutants was fixed in an open position, and this likely resulted in forces being exerted on the basihyal and ceratohyal by the interconnecting muscles during juvenile development. The outcome of these forces varied between different individuals. Fig 6E shows an individual where the ventral position of the lower jaw appeared to have pushed the basihyal and ceratohyal posteroventrally, resulting in a sharp angle between the anteroventrally-pointing anterior end of the ceratohyal and the posteroventrally-pointing posterior end of the basihyal. Other individuals (S3A Fig) had a relatively normal position of the basihyal and ceratohyal in the mouth compared to the ventrally-positioned lower jaw, resulting in the basihyal partially obstructing the open mouth, its anterior end positioned dorsally to the entire lower jaw. In some mutants (n = 2/5) the basihyal was bent half-way and ossified in an L-shape (S3B Fig).

Viewed anteriorly (Fig 6F–6H), the face of the mutant appeared “pinched” at the position where the jaw joint would have formed, resulting in a reduced area of the mouth opening (n = 5/5). There were also impacts on the bones of the upper jaw, the premaxilla and maxilla, which also appeared to be posteriorly compressed into the cranium and compressed laterally in line with the “pinched” jaw apparatus. As recently described mutant line was reported to lack kinethmoid [9], we segmented the corresponding region and found that the kinethmoid is present in our mutants at both 60 and 90 dpf, and relatively morphologically unchanged as compared to wild-type fish (n = 9/9, S4 Fig).

The cartilaginous symphysis joint between the paired bones of the dentary flexes during feeding in wild-type zebrafish as the jaw is opened and the width of the mouth opening is increased by the lateral flaring of the suspensorium [44,45], illustrated in Fig 6I, 6J, 6L and 6M. In contrast, the symphysis of *nkx3*.*2* mutants tended to be thickened and deformed (n = 5/5, Fig 6K and 6N) possibly leading to reduced flexibility.

### Juvenile and adult *nkx3*.*2*^uu2803/uu2803^ zebrafish display loss of basiventral cartilage and parapophyses

**Fig 7:**
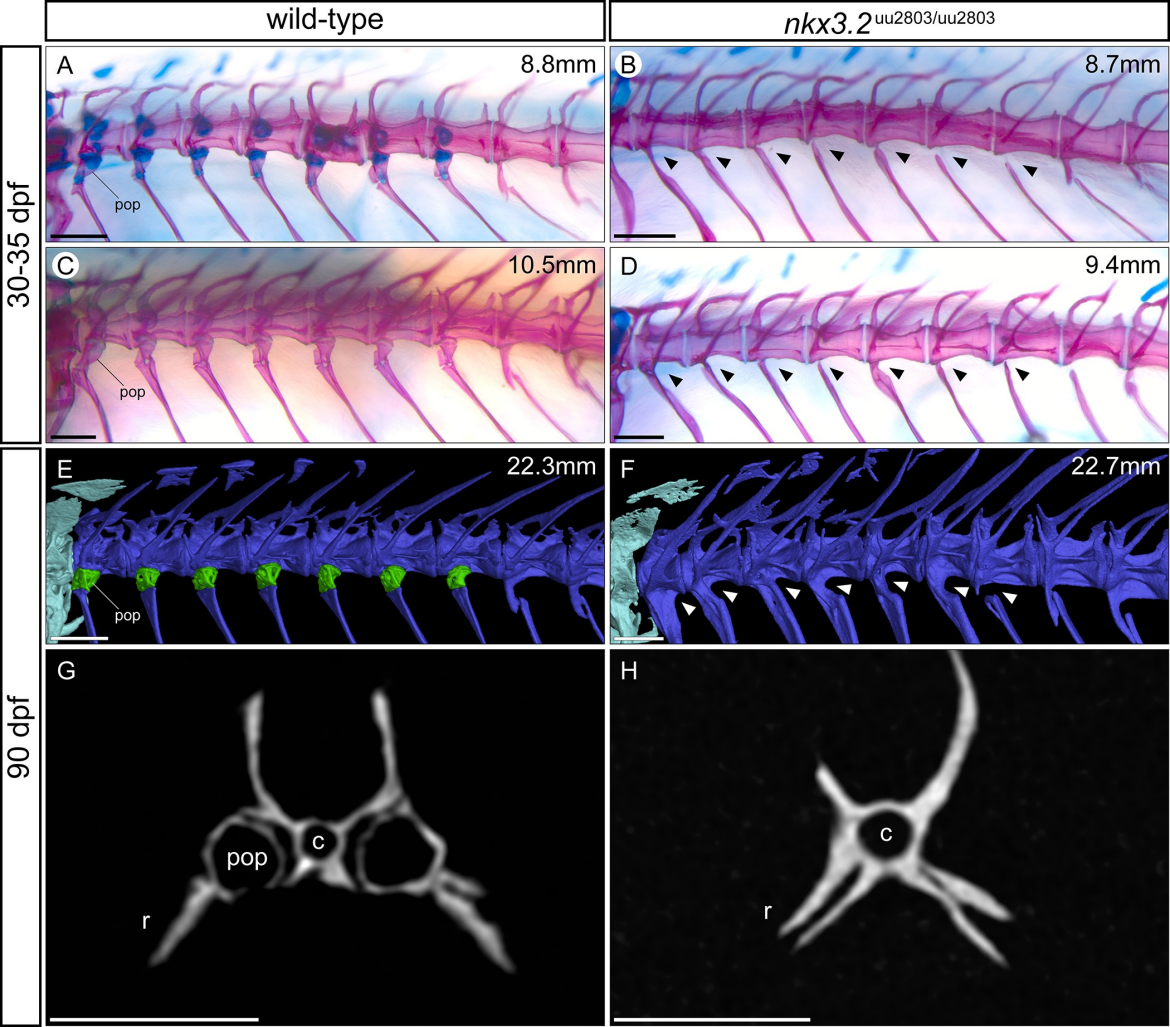
Parapophyses are absent in the rib-bearing vertebrae of *nkx3*.*2*^uu2803/uu2803^ zebrafish. (**A-F**) Dorsolateral views of rib-bearing vertebrae in 30–35 dpf, and 90 dpf wild-type and *nkx3*.*2* mutant zebrafish. (**A-D**) Cartilage- and bone- stained juvenile zebrafish, (**E, F**) μCT models. (**G, H**) μCT virtual transverse cross-sections of wild-type and *nkx3*.*2* mutant rib-bearing vertebrae at 90 dpf. The measurements in mm refer to standard length (SL). Arrowheads in (**B, D, F**) indicate the absence of parapophyses on the rib-bearing vertebrae. Parapophyses (pop) are highlighted in green in (**E**). c–centrum, r–rib. Scale bars: 200μm(**A-D**), 500μm (**E-H**).

Next, we assessed the effect of *nkx3*.*2* loss-of-function on the axial skeleton of juveniles and adults. Mutants at 8.7 mm SL displayed a loss of basiventral cartilage in the precaudal rib-bearing vertebrae (Fig 7B) compared to wild-types of 8.8 mm SL (Fig 7A). Wild-type fish at 10.5 mm SL also lacked these cartilages (Fig 7C), as they have been entirely replaced through endochondral ossification by the parapophyses, small articulating bones that connect the ribs to the vertebrae. However, in 30–35 dpf *nkx3*.*2* mutants (8.7–9.4 mm SL), it was clear that this endochondral ossification of basiventral cartilage had not taken place, as the parapophyses were absent (n = 4/4, Fig 7B and 7D). Instead, the ribs were fused directly to the vertebrae without any articulating process, while some other (more posterior) ribs were entirely disconnected from the vertebrae. This phenotype persisted in 90 dpf *nkx3*.*2* mutants, as seen in μCT segmented models and virtual histological sections of the vertebrae (Fig 7E–7H). In contrast, the neural arches, zygapophyses, and haemal arches appeared to develop normally (n = 4/4).

### OPT, skeletal staining, and μCT reveal changes in the occiput and Weberian apparatus of *nkx3*.*2*^uu2803/uu2803^ zebrafish

Comparison of OPT reconstructions of 5 dpf larvae (Fig 4) suggested the reduction of the cartilage of the auditory capsule changing the shape of the notochord insertion area in *nkx3*.*2* mutants. At 30 dpf cartilage and bone staining revealed changes in the occipital region and cervical vertebrae in *nkx3*.*2* mutant compared to wild-type zebrafish. More specifically, *nkx3*.*2* mutants displayed smaller lateral occipital fenestrae, due to changes in the shape of the exoccipital and supraoccipital as well as reduced basiventral cartilages and cartilages associated with forming Weberian apparatus (Fig 5G” and 5H”).

**Fig 8:**
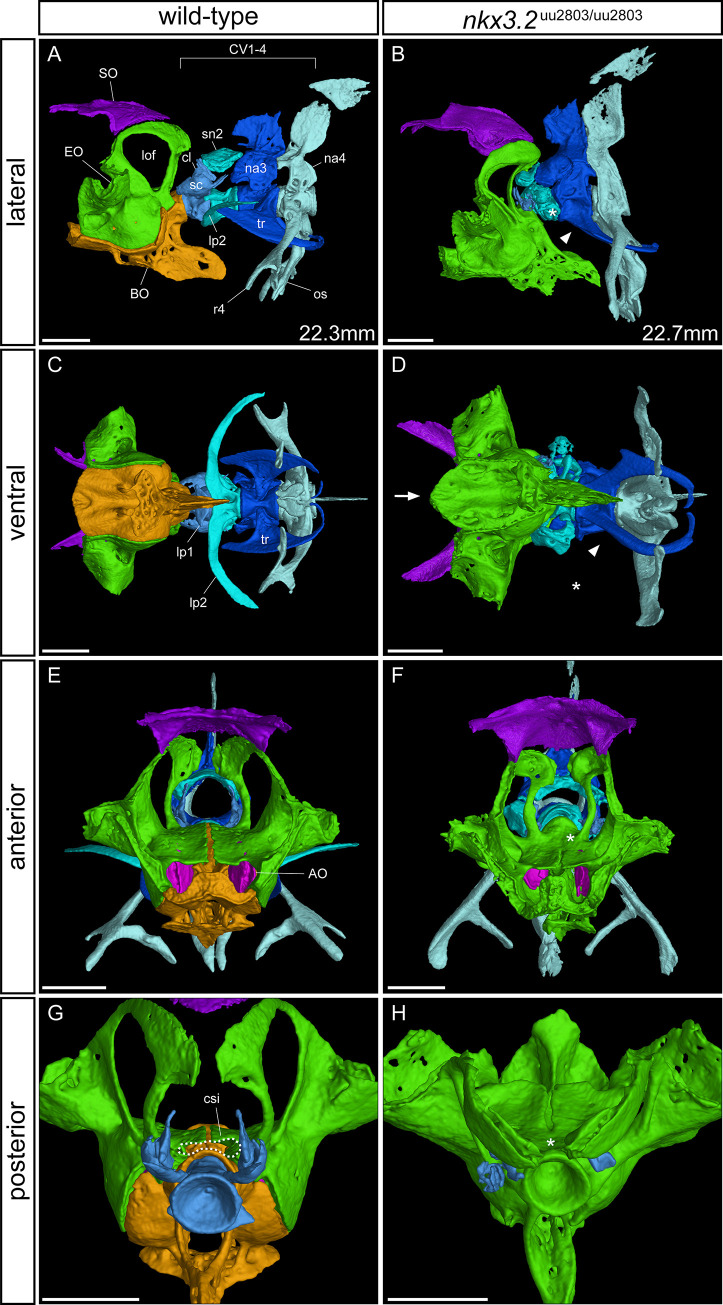
μCT reveals phenotypes in the occiput and Weberian apparatus of *nkx3*.*2*^uu2803/uu2803^ zebrafish. Lateral, ventral, anterior, and posterior views of wild-type (**A, C, E, G**) and homozygous mutant (**B, D, F, H**) occiput and Weberian apparatus. Cervical vertebrae (CV) 2–4 have been removed in (**G, H**) for clarity. Asterisk in **B**, **D** indicates the absence or severe reduction of lateral process 2 from CV2. Arrowhead in **B**, **D** indicates the absence of anterior ramus of tripus on CV3. Arrow in **D** indicates the V-shaped anteroventral edge of basioccipital. Asterisk in **F** indicates the posterior fusion of the medial gap between the paired exoccipital bones. Dotted line in **G** highlights the cavum sinus impar (csi), while the asterisk in **H** indicates its absence. The measurements in mm refer to standard length (SL). AO–asteriscus otolith, BO–basioccipital, csi—cavum sinus impar, cl–claustrum, EO–exoccipital, lof–lateral occipital fenestrae, lp–lateral process, na–neural arch, os–os suspensorium, r4 –rib 4, sc–scaphium, sn2 –supraneural 2, SO–supraoccipital, tr–tripus. Scale bars: 500μm.

To investigate these phenotypes in greater detail, these bones were segmented from μCT scans of 90 dpf wild-types and *nkx3*.*2* mutants. In the occipital region at the back of the skull, mutants displayed a dramatic fusion between the basioccipital and exoccipital (n = 5/5, wild-type is shown in Fig 8A, 8C, 8E and 8G), making it impossible to clearly demarcate these two elements, hence they are the same colour in mutants displayed in Fig 8B, 8D, 8F and 8H. The medial gap between the paired exoccipital bones (Fig 8E) was also partially or completely fused in mutants (Fig 8F). As a result of this fusion and the fusion between the exoccipital and basioccipital, the posterodorsal opening of the cavum sinus impar (csi) was lost or greatly deformed (Fig 8G and 8H). The anteroventral surface of the basioccipital that contacts the parasphenoid and prootic was highly convex in mutants, compared to only slightly convex in wild-types (Figs 8C, 8D and S5). The posterodorsal exoccipital struts were impacted by the cervical vertebrae in *nkx3*.*2* mutants (n = 5/5, Fig 8B), causing the lateral occipital fenestrae to be reduced in area and rotated posteromedially.

Wild-type zebrafish possess four distinct cervical vertebrae (CV), while most mutants (n = 4/5) lacked the first and most anterior CV (Fig 8A and 8B), with one mutant also missing CV2. Dorsal to CV1 in wild-type zebrafish are the scaphium and claustrum, which were entirely lost or highly reduced in all mutants lacking CV1 (Fig 8A, 8B, 8G and 8H). The lateral process (lp2) on CV2 was significantly reduced in mutants, varying between individuals from a ~50% reduction in size to almost complete loss (Fig 8C and 8D). The tripus of CV3 was deformed in mutants—the anterior ramus and articulating process were absent (Fig 8A–8D). The articulation of rib 4 and the os suspensorium to CV4 was also altered in mutants, consistent with the absence of parapophyses observed in the rib-bearing vertebrae of vertebrae 5–11 (Fig 7E and 7F).

## Discussion

In this study, we generated a novel zebrafish null mutant of *nkx3*.*2*. Remarkably, and consistent with other *nkx3*.*2* mutant alleles [9,26], zebrafish homozygous for this mutation survived until adulthood, allowing us to study the mutant phenotype at a range of developmental stages up to and including adults. We employed traditional and novel techniques to characterise the effects on skeletal development caused by this mutation. Consistent with previous studies, we identified a key role for *nkx3*.*2* in the development of the jaw joint. However, we also described axial phenotypes that are novel in zebrafish, although they closely mirror phenotypes reported in human and mouse.

Homozygote *nkx3*.*2* mutants displayed a prominent open mouth phenotype from 14 dpf onwards. We did not perform experiments to study the feeding behaviour, but a recent study analysing the open mouth phenotype found that *nkx3*.*2* mutants employ ram feeding [9]. This phenotype in our *nkx3*.*2*^-/-^ zebrafish is caused by loss of the jaw joint and as a result, the fusion between Meckel’s and palatoquadrate cartilages of the first pharyngeal arch and loss of the retroarticular process (RAP). Our findings are consistent with the previous studies showing expression of *nkx3*.*2* in the jaw joint of zebrafish [3,46], knockdown of *nkx3*.*2* in *Xenopus* [8], and both knockdown and null mutants in zebrafish that result in the fusion of the jaw joint articulating elements Meckel’s cartilage and palatoquadrate accompanied by the loss of the RAP [3,9,26]. These results clearly show the importance of Nkx3.2 during primary jaw joint development. Nkx3.2 loss in mouse does not affect the jaw joint but rather the associated bones of the homologous structures present in the middle ear [13].

Our results are in agreement with findings from previous studies describing Nkx3.2 as a chondrocyte maturation inhibiting factor during skeletogenesis, which is able to repress the skeletogenesis factor Runx2 [18,19]. As fused Meckel’s and palatoquadrate cartilages in *nkx3*.*2* mutants displayed the alignment of chondrocytes and their ability to become hypertrophic throughout the fused first arch elements. Moreover, in adult mutant zebrafish, fused articular-quadrate displayed typical characteristics of endochondrally ossified spongy bones (Fig 3F’) characterized by adipocytes present in the interspace between the trabeculae [47]. It is also possible that loss of Nkx3.2 could affect another transcription factor Barx1. In contrast to Nkx3.2, Barx1 functions in repressing joint formation and promoting cartilage development. It is expressed in the first pharyngeal arch sub-intermediate domain in wild-type zebrafish, in the cartilage distal to the jaw joint [48]. It would be interesting to investigate whether loss of Nkx3.2 could allow the expansion of *barx1* expression domain, reinforcing the loss of joint identity in the region. The future examination of altered gene expression patterns of chondrogenic and joint specific factors in *nkx3*.*2* mutant zebrafish will be beneficial for understanding the jaw joint establishment process during development.

In addition to the primary jaw joint, *nkx3*.*2 (bapx1)* is also expressed in the symphysis joint in zebrafish embryos [3,46]. Optical projection tomography of 5 dpf mutant larvae detected a change in the shape and increased thickness of the anterior Meckel’s cartilage (Fig 4G–4I). In addition, our μCT analysis of the bones of 90dpf adult mutants showed a prominent thickening and deformity of the symphysis potentially leading to jaw inflexibility (Fig 6K and 6N). Potentially, our results could indicate that Nkx3.2 has a role in shaping the symphysis during zebrafish development.

Similarly, a second arch midline domain corresponding to basihyal was previously reported as expression site of *nkx3*.*2 (bapx1)* [3,46] and it displayed both size reduction and down-regulation of *gdf5* expression in zebrafish *nkx3*.*2* knockdown experiments [3]. Our results indicated that position of the basihyal and ceratohyal, as well as the shape of the basihyal itself was variable in adult *nkx3*.*2* mutant zebrafish (Figs 6 and S4). As there are several craniofacial muscles interconnecting Meckel’s, basihyal and ceratohyal cartilages [49], most probably these are affected by the jaw joint fusion leading to the fixed open mouth phenotype. As a result, the position of the basihyal and ceratohyal, and possibly also the shape of the basihyal, is affected in our *nkx3*.*2* mutants. However, previously reported expression in basihyal and its reduction in *nkx3*.*2* knockdown zebrafish indicated that *nkx3*.*2* is necessary for basihyal development [3]. Therefore we could suggest that these morphologies may have a dual origin as the result of phenotypic accommodation of the open mouth phenotype [9] and a direct effect of the loss-of-function mutation. In order to conclude on the role of *nkx3*.*2* in shaping the symphysis and basihyal, it would be beneficial to investigate any differential expression of relevant genes in these structures in *nkx3*.*2* mutant zebrafish in the future.

In addition to confirming the essential role of Nkx3.2 in the development of the jaw joint, we describe previously unexplored axial phenotypes associated with *nkx3*.*2* loss-of-function in juveniles and adults. *nkx3*.*2* expression has previously been described around the anterior notochord, in median fins, in neural arches, hemal arches, and spines of the vertebrae [28], but our analysis of *nkx3*.*2*^-/-^ phenotypes sheds more light on its specific axial role.

The parapophyses that articulate ribs 5–11 with the vertebral centra were absent in juvenile and adult mutants, with many ribs instead ossifying directly onto the centra, and some remaining entirely disconnected from the centra, separated by a gap where a parapophysis would have been located (Fig 7). The paired parapophyses normally ossify endochondrally from basiventral cartilages ventrolateral to each centrum, whereas in *nkx3*.*2* mutants it appears that this cartilage either fails to properly form or is rapidly lost. This basiventral phenotype is reminiscent of the vertebral defects identified in mouse mutants, namely that the ventromedial vertebral ossification centres fail to form [15,16,50]. Similar defects in vertebral ossification have been identified in human patients suffering from SMMD, a disease caused by inactivating mutations in *NKX3*.*2* [21–25].

A major difference between the centra of teleosts and mammalian tetrapods is that the former directly ossify into bone without a cartilage precursor [51], while the latter ossify endochondrally [52]. The cartilaginous vertebral precursors in tetrapods are derived from the sclerotome, while in zebrafish the notochord sheath mineralises to form chordacentra, followed by a sclerotome-derived intermembranous bone, forming autocentra [51,53,54]. Chondrichthyans and other non-tetrapod osteichthyans also form cartilaginous anlage of vertebral bodies, indicating that teleosts represent a derived condition [52,55–58]. The basiventral and basidorsal cartilage elements in teleosts are derived also from the sclerotome [59,60]. Our results are consistent with a role for Nkx3.2 in sclerotome-derived cartilage development in osteichthyans, rather than in vertebral development more generally. Thus, vertebrae-associated cartilages are affected in some way in the zebrafish, mouse, and human, but lead to different specific defects in teleosts compared to mammals as a result of these fundamental differences in vertebral development. Similar skeletal phenotypes are observed in knockouts of other transcription factors known to be involved in sclerotome patterning, such as Pax1 [61,62] and Gli2 [63].

Defects in the cervical vertebrae is another shared phenotype caused by *nkx3*.*2* mutations in the zebrafish, mouse, and human. In human SMMD patients, the reduced ossification of cervical vertebrae can lead to kinking of the neck (kyknodysostosis) and secondary neurological problems associated with an injured cervical cord [25]. *Nkx3*.*2* knockout mouse embryos display a lack of chondrogenesis in the cervical vertebral bodies [16,64], and this is also potentially the case in human SMMD embryos, part of the aetiology of the severe cervical defects seen postnatally. In zebrafish and other members of the teleost superorder Ostariophysi, especially otophysians, the cervical vertebrae and their associated elements have a unique structure collectively termed the Weberian apparatus [65]. This complex of bones is adapted for transmitting sound from the swim bladder to the inner ear along a chain of bony elements connected by ligaments. Many of these bony elements represent highly derived cervical ribs and neural arches of vertebrae 1–4 [65]. In *nkx3*.*2* mutant zebrafish, we observed defects in all the ventral elements of the Weberian apparatus: lateral process 2, the tripus, and rib 4/os suspensorium (Fig 8). In the tripus and rib 4, the parts of these elements that are likely derived from basiventral cartilages—the anterior ramus of the tripus and both articulating processes [65]—are absent or malformed such that the tripus and rib 4 are fused to cervical vertebrae 3 and 4 respectively, reminiscent of the phenotype in the other rib-bearing vertebrae as a result of the absence of parapophyses. Lateral process 2 on CV2 is absent or highly reduced in mutants. Dorsal elements of the Weberian apparatus, on the other hand, such as the supraneurals and neural arches, appear relatively unaffected. However, it is difficult to interpret the direct effects on the scaphium and claustrum in *nkx3*.*2* mutants because of the common loss of CV1 and the space dorsal to it that we suspect results from the impaction of the cervical vertebrae into the occiput. These results further support an essential role of Nkx3.2 that is restricted to the basiventral and not the basidorsal cartilage in zebrafish. Recently described nkx3.2 mutant line displayed shortening of the rostral spine and misalignment of ribs [26], however a detailed description of the affected cervical and vertebrae associated structures was lacking making it difficult to precisely compare the phenotypes with our mutant.

Basiventral cartilage elements were once thought to be a gnathostome-specific feature until it was revealed that hagfish, one of the two extant agnathan vertebrate taxa, possessed basiventral elements [66]. The lamprey, on the other hand, only possesses basidorsal elements, leading to the hypothesis that this taxon secondarily lost basiventral elements and that the ancestral vertebrate possessed both basiventral and basidorsal elements [66], although it is also possible that these elements evolved multiple times independently. Studies on the potential involvement of Nkx3.2 in the development of hagfish basiventral elements may shed light on its potentially pivotal role in this important vertebrate innovation—was it essential in early vertebrates, was it only recruited later in the gnathostome lineage, or could it have been co-opted in basiventral cartilage development multiple times independently?

In the occiput of adult *nkx3*.*2* mutant zebrafish, we observed a partial or complete fusion between the bones of the exoccipital and basioccipital that resulted in a partial or complete loss of the cavum sinus impar. Before that, at 30 dpf our stained mutants revealed changes in shapes of occipital cartilages and smaller lateral occipital fenestrae (Fig 5H”). Already at 5 dpf our OPT results revealed a subtle but significant reduction in cartilage staining intensity in this region (Fig 4), which would have gone unnoticed comparing individual images of the larvae by eye. These results highlight the utility of the OPT method in identifying subtle phenotypes in larval stages, especially in cases where the characterisation of adult phenotypes may not be possible. In mouse knockouts, these same occipital bones are misshapen and underdeveloped [15,16,50], although fusions between them have not been described. In addition, these *Nkx3*.*2* knockout mice display an absence of the supraoccipital bone, while the supraoccipital in zebrafish appears to develop normally. There are no reports of any defects to the occipital bones of human SMMD patients, although it is not clear whether this is because no defects exist or because they have been overlooked, likely a result of the difficulty in studying affected individuals *in utero* or shortly after birth.

In gnathostomes, the most anterior somites contribute to the occipital bones [67–69]. The sclerotome from these occipital somites contributes to the basioccipital and exoccipital [70–72], so the occipital phenotypes observed in zebrafish and mouse *Nkx3*.*2* mutants are consistent with the role of Nkx3.2 in the sclerotome. The combination of these occipital phenotypes, particularly the defects in the cavum sinus impar and Weberian apparatus suggest that *nkx3*.*2* mutant zebrafish should have a severe hearing impairment, although we did not test this.

*Nkx3*.*2* expression has previously been identified in the median fins of zebrafish [28] and the limb buds and digits of mice [14,16,50]. Like mammalian limb bones, teleost median proximal radials first develop as hyaline cartilage before endochondral ossification takes place [73,74]. Human SMMD patients often display several limb defects postnatally: long limbs, the presence of large epiphyses and irregular metaphyses that give the disease its name, and the presence of pseudoepiphyses in the digits combined with reduced ossification of the carpals [21,23–25]. Limb defects have not been reported in mouse mutants [15,16,64], but downregulation of *Nkx3*.*2* expression has been linked to increased tibia lengths [75] while overexpression causes the opposite–skeletal dwarfism [76]. These studies suggest that the gene has a similar role in humans and mice and that the reason limb defects have not been found in mouse mutants is that the mutation is perinatally fatal, while these limb phenotypes appear postnatally [21]. These mammalian data indicate a role for Nkx3.2 in repressing chondrocyte maturation and therefore endochondral bone formation.

Our study was not designed to specifically investigate the effects of *nkx3*.*2* loss-of-function on zebrafish median fins, and we could not detect any significant and consistent changes to these bones with the data we have available. Previous studies investigating other *nkx3*.*2* mutant zebrafish lines also did not describe any median fin phenotypes [9,26]. The proximal radials of the dorsal and anal median fins develop with contributions from somite-derived cells including the sclerotome [77,78], while paired fins and limbs are derived from the lateral plate mesoderm [78,79]. Even though these different skeletal structures develop from different progenitor populations, it has long been recognised that the paired limb buds redeploy some developmental mechanisms that first evolved in the median fins [77]. It may be that the function of Nkx3.2 diverged in fins and limbs following that redeployment, although a more detailed study of the role of Nkx3.2 specifically in median fin development is required to test this hypothesis.

This study highlights the role of *nkx3*.*2* in the development of different skeletal tissues formed on the base of cartilage template. In the jaw mutant phenotypes are consistent with a loss of chondrocyte maturation inhibition, while in the basiventral cartilages along the precaudal vertebrae, Nkx3.2 appears to be required for the onset or maintenance of cartilage development. Therefore, the role of Nkx3.2 in promoting and maintaining chondrocyte proliferation is of interest to investigate in the future, until now only few reports have been focusing on this function of Nkx3.2 [80]. Comparing our results with studies of amniote model systems and human disease reveal a largely consistent function of this gene between teleosts and amniotes, suggesting their inheritance from an early osteichthyan ancestor. Future studies in chondrichthyans and agnathans will further inform our understanding of the function of Nkx3.2 in early vertebrate evolution.

## Supporting information

S1 Fig Standard lengths (SL) of a sample of wild-type and mutant zebrafish at 30, 60, and 90 dpf. *nkx3*.*2* mutant zebrafish display variable body sizes at different developmental stages. SL measurements were made according to Parichy *et al*. [81]. The differences in SL between wild-type and mutant groups were apparent at 30 dpf and 60 dpf. Possible explanations are that bigger SL in mutants at 30 dpf was due to overeating caused by constant swimming through food with an open mouth, whereas smaller SL at 60 dpf was due to some competition for food before separation of the mutant group at 60 dpf.
(TIF)

S2 Fig Voxel-wise method is robust to false positives. (**A, B, C**) Maximum projection of both the 5 dpf wild-type (n = 10) and *nkx3*.*2* mutant (n = 11) groups, with coloured voxels representing voxels with statistically significant (p<2.5x10^-5^) differences in intensity. Cyan shows voxels with higher intensity in wild-type group and magenta shows voxels with higher intensity in *nkx3*.*2* mutant group. (**D, E, F**) The same analysis performed using a randomised subset of larvae instead of comparing wild-type and *nkx3*.*2* mutant groups. The absence of cyan and magenta voxels indicates a lack of statistically significant false positives in the comparison of these randomised groups. Scale bars: 150μm.
(TIF)

S3 Fig Basihyal shape and position is highly variable in *nkx3*.*2* mutants. (**A, B**) Two examples of additional 90 dpf mutant craniofacial phenotypes in addition to that shown in Fig 6. (**A**) The basihyal (BH) and ceratohyal (CH) are positioned as in wild-types relative to the rest of the head, resulting in an obstruction to the open mouth resulting from the jaw joint fusion. (**B**) The basihyal is ossified into an L-shape. Scale bars: 1mm.
(TIF)

S4 Fig Kinethmoid bone is present in adult *nkx3*.*2* mutants. (**A-D**) Dorsal view of isolated upper jaw elements coloured by light blue–premaxilla, pink—maxilla (mx), green—kinethmoid (k), yellow—preethmoid (pe), dark blue—palatine (pa), and orange—ethmoid (e). (**A**, **B**) 60 dpf wild-type (artificially open mouth) and *nkx3*.*2* mutant, respectively. (**C**, **D**) 90 dpf wild-type (artificially open mouth) and *nkx3*.*2* mutant, respectively. Scale bars: 200μm.
(TIF)

S5 Fig Ventral views of the adult skull. 90 dpf wild-type (**A**) and *nkx3*.*2* mutant (**B**). Mutants display a reduced area of the optic foramen as a result of posteromedial expansion of the orbitosphenoid and pterosphenoid (arrowheads). The anterior edge of the ventral surface of the basioccipital is V-shaped as it meets the parasphenoid (arrow). BO–basioccipital, EO–exoccipital, le–lateral ethmoid, obs–orbitosphenoid, of–optic foramen, pro–prootic, ps–parasphenoid, pts–pterosphenoid. Scale bars: 1mm.
(TIF)

S1 Movie Larval development of wild-type and *nkx3*.*2*^uu2803/uu2803^ jaw joints seen in Z-stacks. (**A-H**) Movies moving back and forth though Z-stacks of confocal live imaging Z-stacks acquired from ventral side of wild-type zebrafish head at 3 dpf (**A**), 5 dpf (**B**), 7dpf (**C**), 14 dpf (**D**) and *nkx3*.*2*^uu2803/uu2803^ zebrafish head at 3 dpf (**E**), 5 dpf (**F**), 7 dpf (**G**), 14 dpf (**H**) in Tg(*sox10*:*egfp*) background. Scale bars: 25 μm.
(MOV)

S1 Data Data for S1 Fig. (CSV)
