# Supplementary figures and images for: The broad role of Nkx3.2 in the development of the zebrafish axial skeleton
Source: PLoS One. 2021 Aug 19;16(8):e0255953. doi: 10.1371/journal.pone.0255953 (PMC8376051; doi:10.1371/journal.pone.0255953)

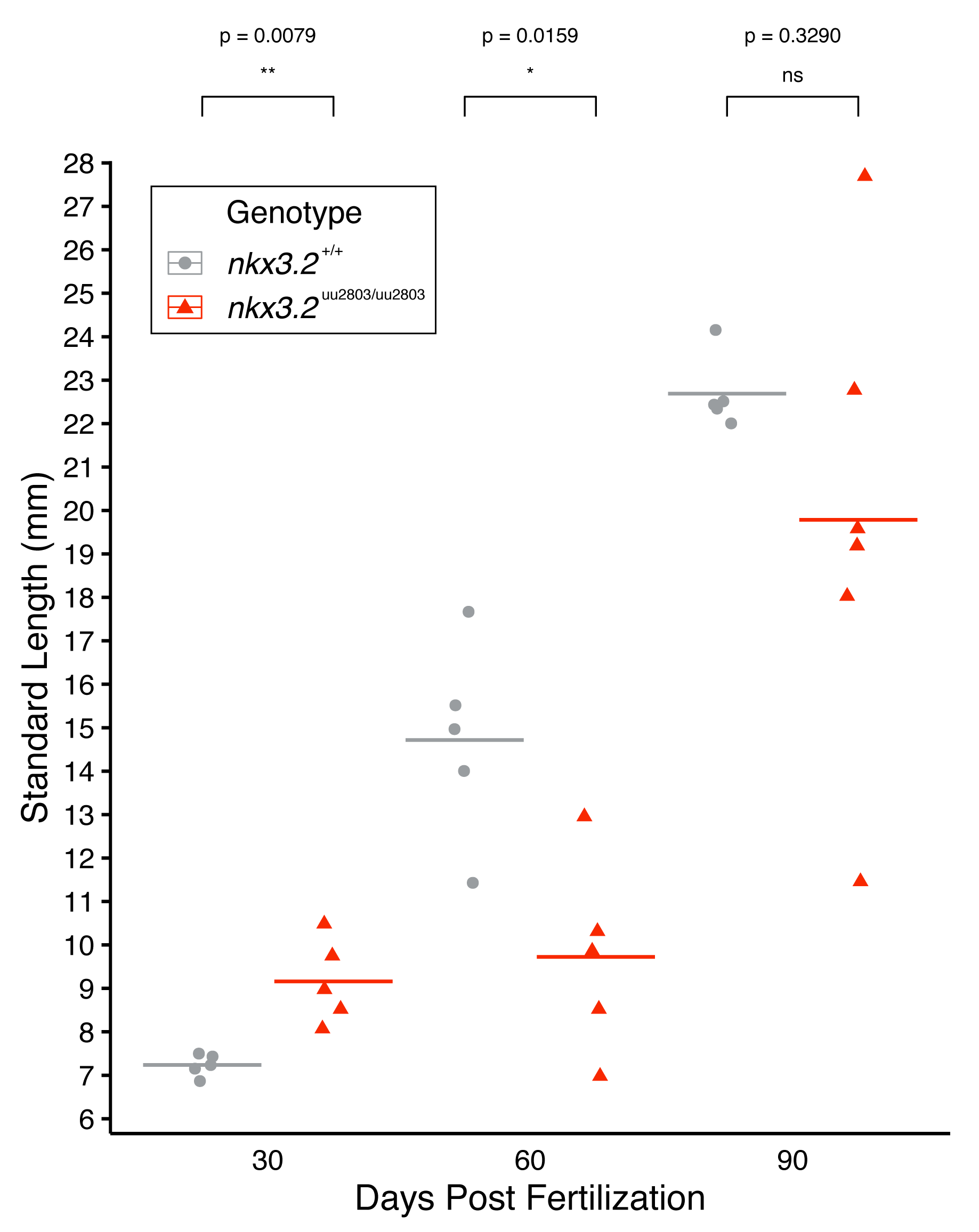

Supplement: S1 Fig — nkx3.2 mutant zebrafish display variable body sizes at different developmental stages. SL measurements were made according to Parichy et al. [81]. The differences in SL between wild-type and mutant groups were apparent at 30 dpf and 60 dpf. Possible explanations are that bigger SL in mutants at 30 dpf was due to overeating caused by constant swimming through food with an open mouth, whereas smaller SL at 60 dpf was due to some competition for food before separation of the mutant group at 60 dpf. (TIF) [file pone.0255953.s001.tif]

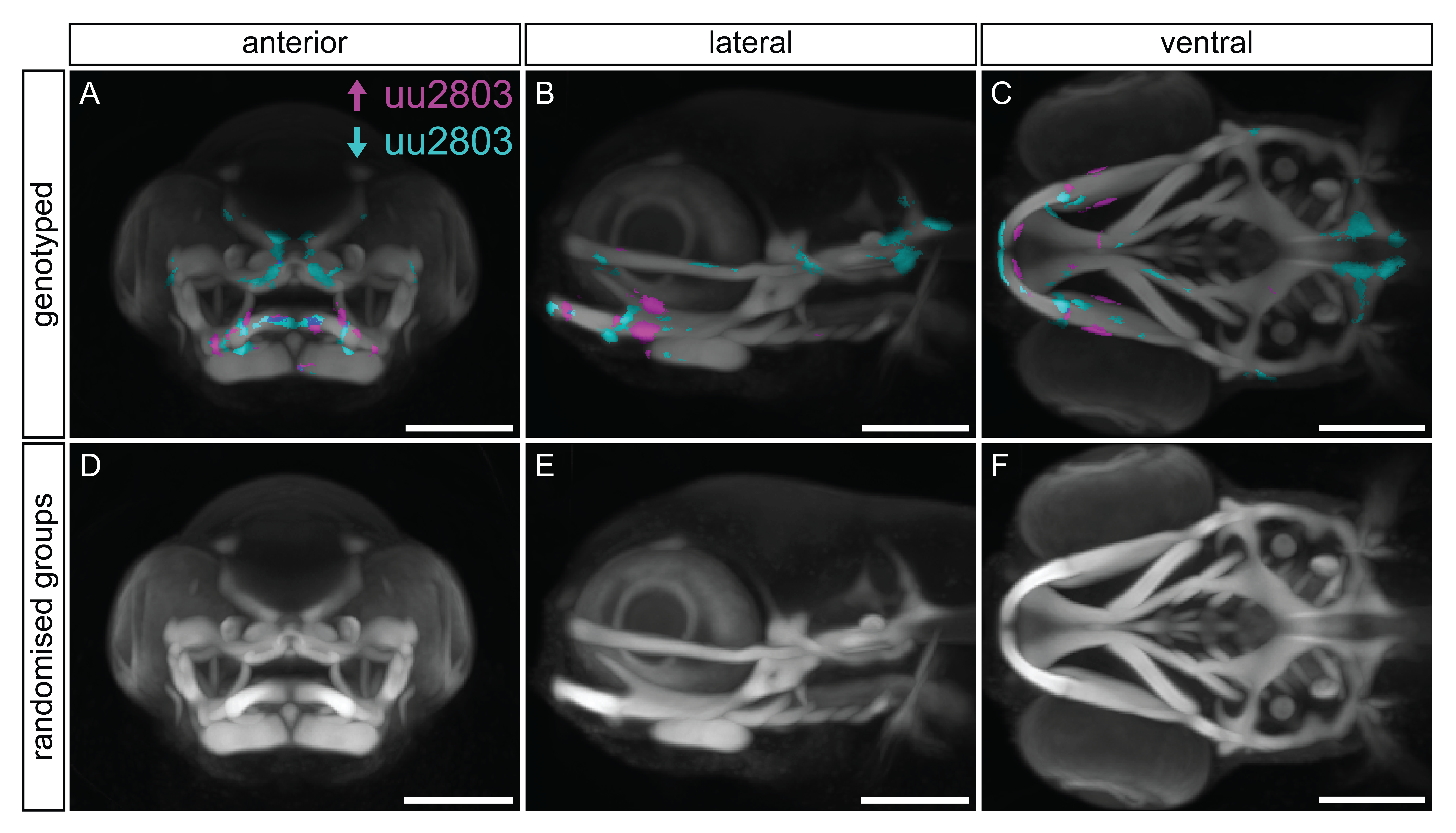

Supplement: S2 Fig — (A, B, C) Maximum projection of both the 5 dpf wild-type (n = 10) and nkx3.2 mutant (n = 11) groups, with coloured voxels representing voxels with statistically significant (p<2.5x10^-5^) differences in intensity. Cyan shows voxels with higher intensity in wild-type group and magenta shows voxels with higher intensity in nkx3.2 mutant group. (D, E, F) The same analysis performed using a randomised subset of larvae instead of comparing wild-type and nkx3.2 mutant groups. The absence of cyan and magenta voxels indicates a lack of statistically significant false positives in the comparison of these randomised groups. Scale bars: 150μm. (TIF) [file pone.0255953.s002.tif]

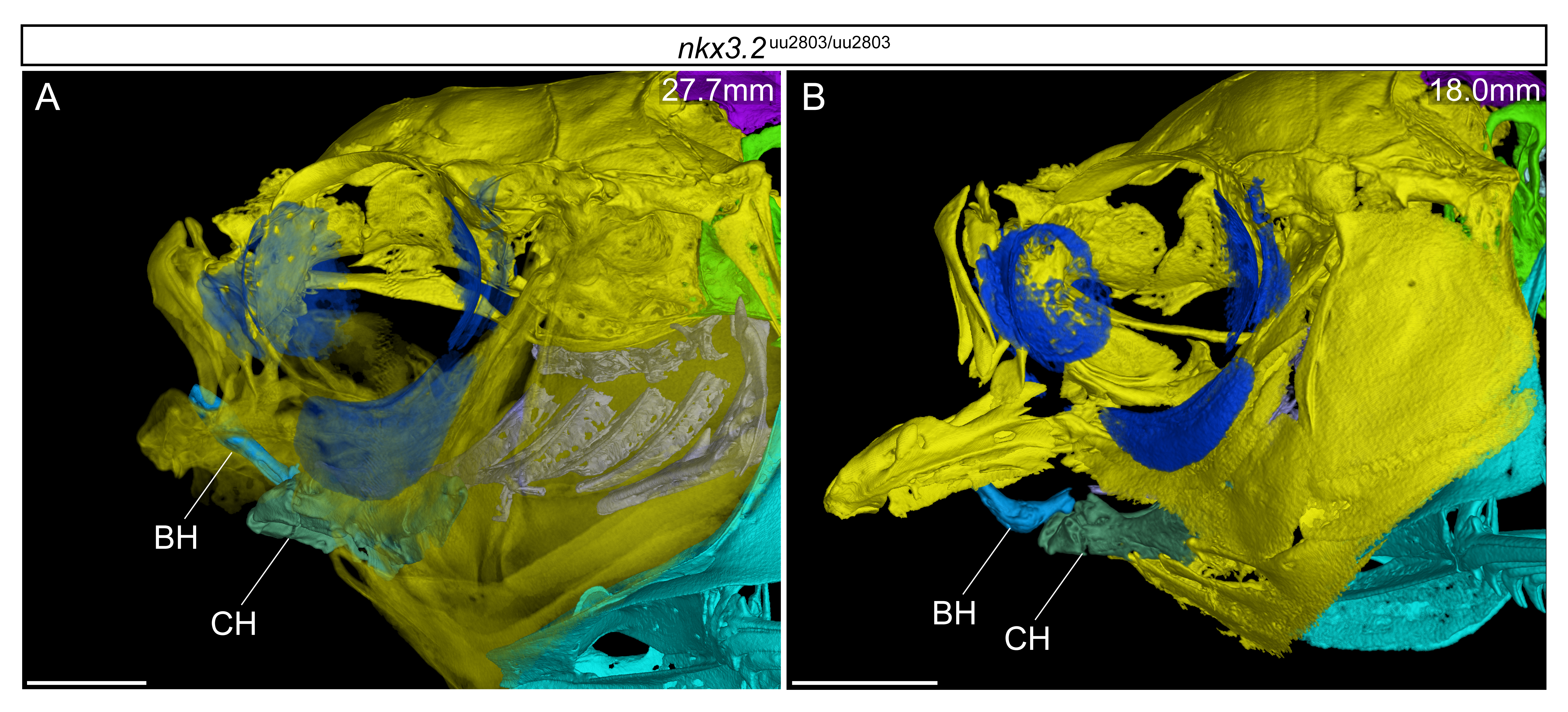

Supplement: S3 Fig — (A, B) Two examples of additional 90 dpf mutant craniofacial phenotypes in addition to that shown in Fig 6. (A) The basihyal (BH) and ceratohyal (CH) are positioned as in wild-types relative to the rest of the head, resulting in an obstruction to the open mouth resulting from the jaw joint fusion. (B) The basihyal is ossified into an L-shape. Scale bars: 1mm. (TIF) [file pone.0255953.s003.tif]

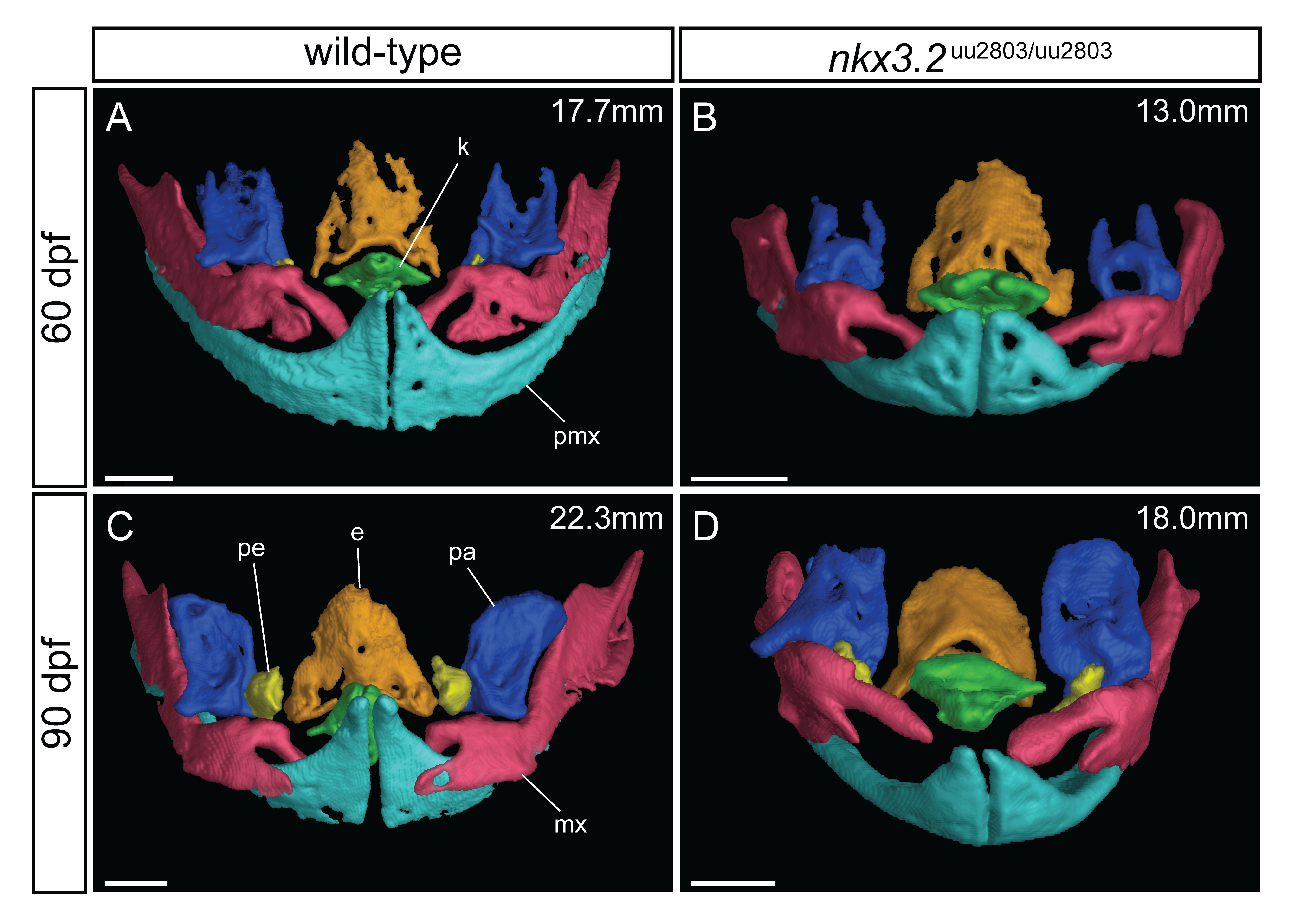

Supplement: S4 Fig — (A-D) Dorsal view of isolated upper jaw elements coloured by light blue–premaxilla, pink—maxilla (mx), green—kinethmoid (k), yellow—preethmoid (pe), dark blue—palatine (pa), and orange—ethmoid (e). (A, B) 60 dpf wild-type (artificially open mouth) and nkx3.2 mutant, respectively. (C, D) 90 dpf wild-type (artificially open mouth) and nkx3.2 mutant, respectively. Scale bars: 200μm. (TIF) [file pone.0255953.s004.tif]

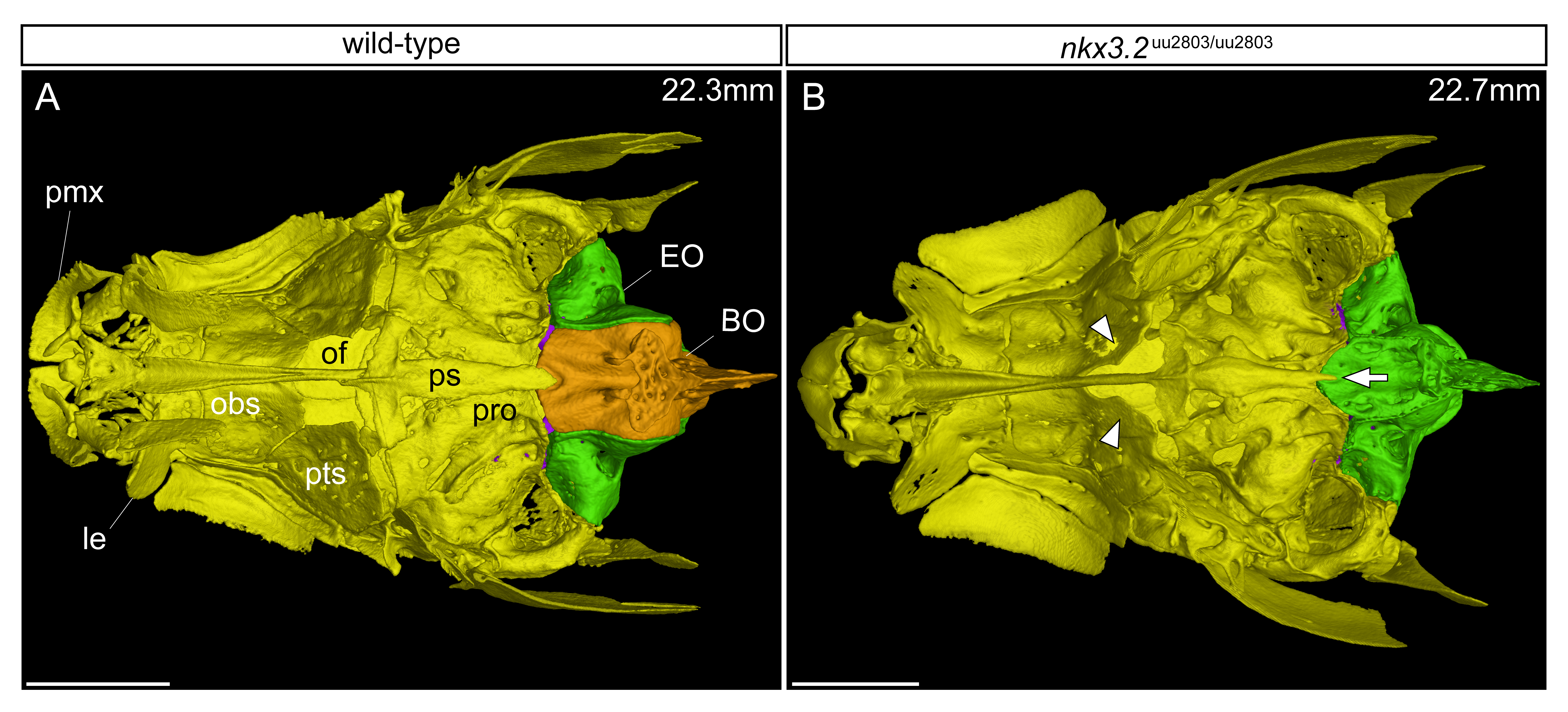

Supplement: S5 Fig — 90 dpf wild-type (A) and nkx3.2 mutant (B). Mutants display a reduced area of the optic foramen as a result of posteromedial expansion of the orbitosphenoid and pterosphenoid (arrowheads). The anterior edge of the ventral surface of the basioccipital is V-shaped as it meets the parasphenoid (arrow). BO–basioccipital, EO–exoccipital, le–lateral ethmoid, obs–orbitosphenoid, of–optic foramen, pro–prootic, ps–parasphenoid, pts–pterosphenoid. Scale bars: 1mm. (TIF) [file pone.0255953.s005.tif]
